# SERS activity of hybrid nano/microstructures Ag-Fe_3_O_4_ based on *Dimorphotheca ecklonis* pollen grains as bio-template

**DOI:** 10.1038/s41598-020-73615-x

**Published:** 2020-10-06

**Authors:** R. D. Ávila-Avilés, N. Torres-Gómez, M. A. Camacho-López, A. R. Vilchis-Nestor

**Affiliations:** 1 Centro Conjunto de Investigación en Química Sustentable (CCIQS), UAEM-UNAM, Km 14.5 Toluca-Atlacomulco Road, CP 50200 Toluca, Estado de México Mexico; 2 Departamento de Genética y Biología Molecular, Centro de Investigación y Estudios Avanzados del Instituto Politécnico Nacional (CINVESTAV-IPN), Av Instituto Politécnico Nacional 2508, CP 07360 La Laguna Ticoman, Ciudad de México Mexico; 3 Tecnológico Nacional de México/Instituto Tecnológico de Toluca, Av. Tecnológico s/n, Colonia Agrícola Bellavista, 52149 Metepec, Estado de México Mexico; 4 Laboratorio de Investigación y Desarrollo de Materiales Avanzados, Facultad de Química, Universidad Autónoma del Estado de México, Campus Rosedal, Km 14.5 Carretera Toluca-Atlacomulco, San Cayetano de Morelos, C.P. 50295 Toluca, Mexico

**Keywords:** Nanobiotechnology, Nanoscale materials, Green chemistry, Materials chemistry

## Abstract

Nature provides remarkable examples of mass-produced microscale particles with structures and chemistries optimized by evolution for particular functions. Synthetic chemical tailoring of such sustainable biogenic particles may be used to generate new multifunctional materials. Herein, we report a facile method for the synthesis of hybrid nano/microstructures Ag-Fe_3_O_4_ based on *Dimorphotheca ecklonis* pollen grains as bio-template. Silver nanoparticles was biosynthesized using pollen grains as a reduction and stabilization agent as well as a bio-template promoting the adhesion of silver nanoparticles to pollen surface. Fe_3_O_4_ nanoparticles were synthesized by co-precipitation method from FeSO_4_. Hybrid nano/microstructures Ag-Fe_3_O_4_ based on *Dimorphotheca ecklonis* pollen grains as bio-template were obtained and characterized using Scanning Electron Microscopy and Transmission Electron Microscopy to study the morphology and structure; Energy-Dispersive X-ray Spectroscopy to determine the chemical composition distribution; and Confocal Fluorescence Microscopy to demonstrate the fluorescence properties of hybrid nano-microstructures. Furthermore, these hybrid nano-microstructures have been studied by Surface-Enhanced Raman Scattering (SERS), using methylene blue as a target molecule; the hybrid nano-microstructures have shown 14 times signal amplification.

## Introduction

A nanostructured hybrid material is a mixture of two or more materials that can express new properties^1^, in which some or all are in the order from 1 to 100 nm. Whereas, a hybrid composite is the combination of a hybrid material with a composite^2^, when that composite presents new functions associated to the mixture of the materials and that are not separately exhibited as a whole, is called hybrid-functional material^3^. All these definitions have been inspired by the developing new materials arrangements with novel properties, which is one of the main objectives for materials science. Throughout this continuous search of new materials, nature is a remarkable source of insption for the design of particles with complex morphologies and intricate structures adapted for a variety of functions^4^. Since the living organisms achieve control at molecular level, bio-inspired design makes it possible to control different aspects in the formation of new materials, including the precise location of the particles, nucleation density, size and stability, furthermore complex architectures can be obtained^5^.

Pollen grains (PG) particles have received considerable attention as template for hybrid structures synthesis because they are abundant, renewable, robust and adhesive microparticles with unique chemical and structural characteristics ^6^. Furthermore, the pollen outer layer’s (called exine) exhibit unusual resistance to degradation by strong chemical regents, this chemical stability is associated with the presence of sporopollenin, one of the most chemically inert biological polymers, as the main component of exine^7^. Additionally, it is possible to select pollen particles with a size of a few to hundreds of microns in nominal diameter, with a remarkable variety of structural patterns from different pollen species^8^. Therefore, in recent years different research groups have developed precise methodologies for he synthesis of metal^9,10^ and oxides^11,12^ nanoparticles using pollen grains as a template. Haisheng Lin et al*.* in 2015 synthesized pollen microparticles with magnetite core^11^; Lucas Johnstone et al*.,* in 2017, reported the synthesis of pollen microparticles decorated with silver nanoparticles and silver-silicon oxide nanoparticles, which showed outstanding optical properties^10^. The usual synthetic methods of hybrid materials based on pollen grains as template, are based on the functionalization of the exine by compounds such as aminopropyl trimethoxysilane (APTES) in strong reaction conditions, but this remains as a challenge due to low chemical reactivity of sporopollenin^11^.

*Dimorphotheca ecklonis* is a plant belonging to the genus *Osteospermum* (Family *Compositae*), formerly *Dimorphotheca*, native to South Africa^13^. *Dimorphotheca ecklonis* is an evergreen sub-shrub characterized by vigorous growth and abundant flowers; It has been converted into an ornamental pot plant having a high economic potential^13^. Studies regarding the formation of the pollen grain of organisms belonging to the genus *Dimorphotheca* revealed that it is characteristic of a reduction in the weight of the pollen grain due to its formation mechanism compared to other species^14^. Similarly, the morphology presented by *Dimorphotheca ecklonis* favors the formation of tectal elements (outermost structure of the pollen grain characterized by a spike or peak shape)^14^. This layer mainly consists of secreted waxes and more volatile lipids as well as flavonoids, steroids, phenolics and aliphatics^7^. The morphology, weight, composition, and size characteristics; also given its ease of cultivation, its global distribution, its high production of flowers and consequently the ease of collecting pollen, it has been chosen in this study as the source of raw material (pollen grains) for development the hybrid nano/microstructures Ag-Fe_3_O_4_.

Surface-enhanced Raman Scattering (SERS) has become an important tool for chemical analysis^15^, single molecule detection^16^, electrochemistry^17^ and biological sensing^18^ due to the high detection limits in different environments without complex preparation requirements of the samples. It is known^19^, that the Raman amplification is closely related to the presence of “hot spots” on the SERS substrate, when some molecule interacts with these “hot spots” induce an enhancement of the Raman signal (SERS). There are different factors that can influence the presence and efficiency of the “hot spots” for Raman signal amplification as: the nature of the metallic nanostructure^20^, the arrangements of the nanoparticles on the substrate^21^, the size^22^ and morphology^23^ of the metallic nanoparticles. The interaction between the tips two nanoparticles have shown the strongest electromagnetic fields amplification, therefore anisotropic metal nanoparticles like cubic, triangular and star shapes usually shows the best SERS response^24^. However, there is other approach to promote the interaction between anisotropic nanoparticles in order to generate active “hot spots”, starting from templates with tips covered with metal nanoparticles and finally assemble in a nanostructured arrangement.

Herein, we report a facile method for the development of hybrid nano/microstructures Ag-Fe_3_O_4_ based on *Dimorphotheca ecklon*is pollen grains as bio-template. This hybrid nanomaterial combines the optical and magnetic properties of Ag and Fe_3_O_4_ nanoparticles respectively, on a complex biogenic structure based on pollen grain in order to generate an opto-magnetic micro-particle substrate for SERS.

## Material and methods

### Materials

Silver nitrate (AgNO_3_), iron (II) sulfate (FeSO_4_), and ammonium hydroxide (NH_4_OH) were obtained from Sigma-Aldrich Chemicals without further purification; deionized water was employed in all the steps of the synthesis. The pollen grains of *Dimorphotheca ecklonis* were collected in Toluca, Estado de México, México, from local producers.

### Synthesis of hybrid nano/microstructures Ag-Fe_3_O_4_

For the synthesis of hybrid nano/microstructures, 10 mg of freshly collected pollen grains (PG), then were suspended in 10 ml of deionized water and dispersed using a vortex stirrer for 5 min finally the PG were recovered by filtration, this washing process was done twice. The washed PG were mixed with 2 ml of a 0.1 M solution of AgNO_3_ and maintained in magnetic stirring for four hours at room conditions. Afterwards the solution was washed using deionized water three times and vacuum filtered using a nitrocellulose filter of 1.2 µm, finally the nano/microstructures were recovered and calcined at 180 °C for 12 h. In order to add magnetic behavior to the biogenic support, the hybrid structures PG-Ag nanoparticles were mixed, with 1 ml of FeSO_4_ 0.1 M, and 18 ml of deionized water under stirring then 1 ml of NH_4_OH was added drop by drop and maintain in agitation for 72 h. Finally, the hybrid nano/microstructures Ag-Fe_3_O_4_ were washed three times using deionized water and vacuum filtered using a nitrocellulose filter (1.2 µm).

### SEM measurements

Hybrid nano/microstructures were mounted on copper tape without coating, for analysis by Scanning Electron Microscopy (SEM) and coupled energy dispersion analysis (EDS). The samples were analyzed using a JEOL JSM-6510LV scanning electron microscope (SEM), with an acceleration voltage of 12 kV, the micrograph was obtained using a secondary electron, in high vacuum mode. Elemental characterization was performed via EDS with a Bruker QUANTAX 200 spectroscope with 129 eV of resolution attached to the scanning electron microscope.

### TEM measurements

In order to characterize the formation of silver nanoparticles on the pollen grains, the nano/microstructure Ag/pollen grain after calcination procedure were infiltrated for 4 h and left overnight only at 100% of the epoxy resin. Embedding was carried out with 100% of the resin and polymerization at 60 ◦C for 24 h. Ultrathin sections were cut using an ultramicrotome (LEICA UC7) and were placed on carbon-coated copper grids.

For Fe_3_O_4_ nanoparticles characterization, five microliters of Fe_3_O_4_ nanoparticles suspension were placed in carbon-coated copper grids and after completely dried; both samples were analyzed under a JEOL-2100 transmission electron microscope (TEM) operated at an accelerating voltage of 200 kV and equipped with two STEM detectors, Bright Field (BF-detector) and Annular Dark Field (ADF-detector). The micrographs collected by TEM were analyzed using the Gatan software Digital Micrograph in order to determine the particle size and morphology. For High Resolution Transmission Electron Microscopy (HRTEM) and Selected Area Electron Diffraction (SAED) analysis, experimental data was indexed on the base of the JCPDS card No. 00-004-0783 of silver and 01-075-1372 for magnetite with an FCC structure.

### Preparation of SERS substrates and SERS measurements

Methylene blue molecule was used as a target to test SERS response. SERS substrates were prepared as follow: Each hybrid nano/microstructures Ag-Fe_3_O_4_ was deposited on glass slides and 5 µl of methylene blue solution (0.012% w/v) was added to each sample, allowing the same to evaporate and thereafter the same volume was added in steps until complete 20 µl. SERS measurements were done with a HR-800 LabRam (Jobin Yvon Horiba) Raman spectrometer. A He–Ne laser (λ = 632,8 nm) was used to excite the methylene blue vibrational modes. The laser power on the sample was 75 µW. All Raman spectra were the average of 10 accumulations of 60 s each one. The Raman range monitored was 400 to 2000 cm^-1^ for all samples.

### Fluorescence analysis by confocal microscopy

The fluorescence properties of the hybrid nano/microstructures were analyzed using a confocal microscope model TCS SPE/CTR 4000 from Leica, equipped with a mercury lamp, a dry-objective (10 ×) and two immersion objectives (40 × and 63 ×, respectively).

## Results and discussion

**Figure 1:**
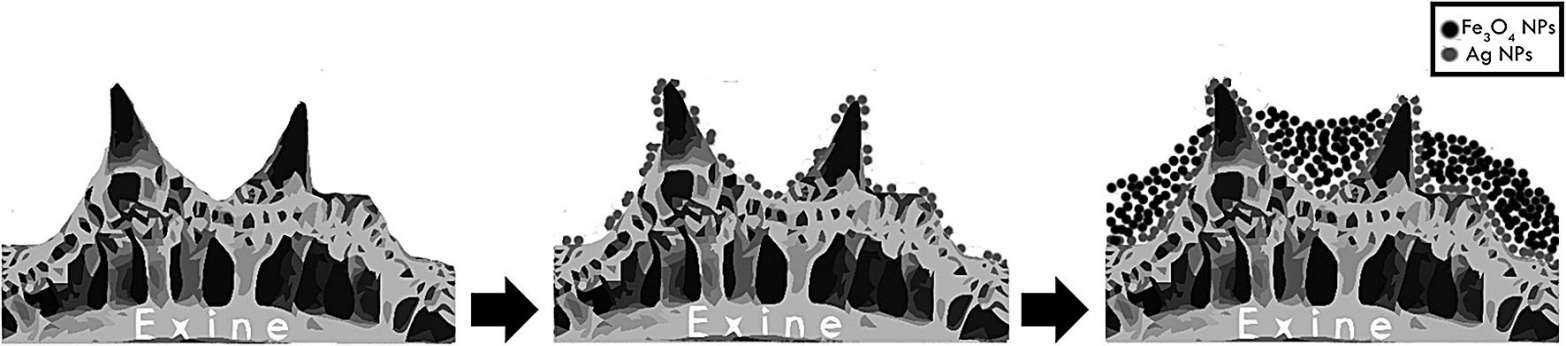
Schematic diagram of synthesis of hybrid nano/microstructures AgNPs-Fe_3_O_4_NPs/PG. (AgNPs in gray and Fe_3_O_4_NPs in black).

The known surface chemical composition and the available range of complex, as well as specific, morphologies of the species make the nanostructured pollen attractive as a microscale bio-template. The use of *Dimorphotheca ecklonis* pollen as a bio-template overcomes the requirement of generate Ag nanoparticles separately as well as eliminate the functionalization stage of the pollen surface since the pollen grains surface of *Dimorphotheca ecklonis* is mainly composed by exine, which contains polyphenols, phenylpropanoids, carotenoids, and fatty acid^25^. These compounds have been reported that can act as bio-reducing and capping agents during the biogenic synthesis of nanoparticles^26–28^. The synthesis of silver nanoparticles through a bioreduction method allows clean, non-toxic, and environmentally friendly synthesis, others that promotes the obtaining of nanoparticles with less cytotoxicity^29,30^. Silver nanoparticles(AgNPs) was obtained by bio-reduction of Ag^+1^ ions with phenolic compounds of the exine, furthermore pollen grains serves as a bio-template since promotes the adhesion of silver nanoparticles (Fig. 1). How is schematized in the Fig. 1 the AgNps are randomly distributed both inside and outside the pollen grain, with a higher prevalence at the tips of the tectal elements.

During the synthesis, the first evidence of the Ag nanoparticles formation was the change of coloration, from yellow color (attributed to the pollen grains) to brown (color of silver nanoparticles) after four hours of synthesis.

**Figure 2:**
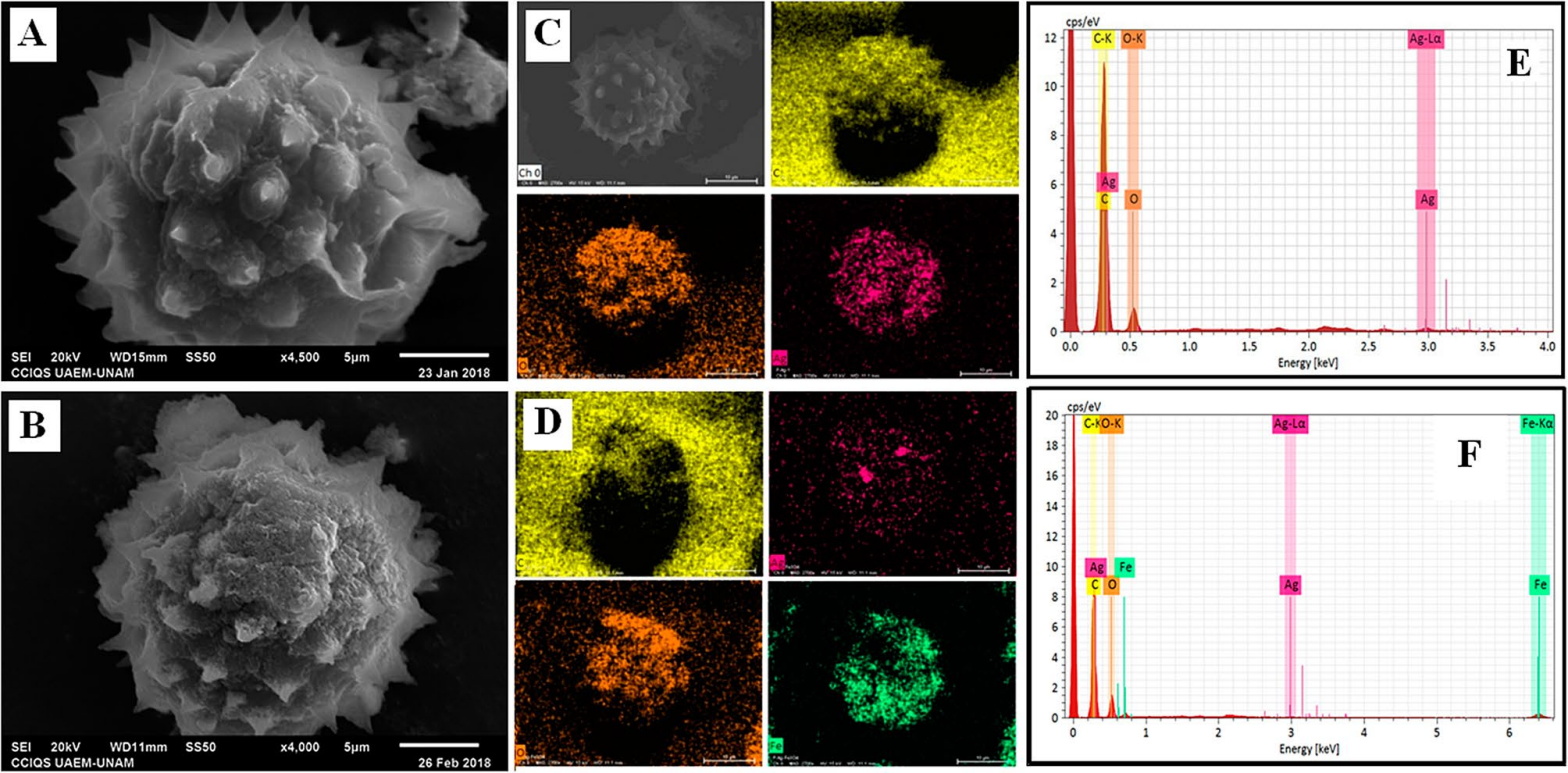
SEM Characterization of nano/microstructures AgNPs/PG **(A)** and hybrid nano/microstructures AgNPs-Fe_3_O_4_NPs/PG **(B)**. Mapping of element distribution and EDS analysis of AgNPs/PG **(C, E)** and AgNPs-Fe_3_O_4_NPs/PG **(D, F)**.

SEM measurements and EDS analysis show that the functionalization of pollen grains was successful and do not show changes on morphology of the pollen grain (Fig. 2A); also, the mapping and EDS show a homogenous distribution of the AgNPs under the template and the presence of the elements carbon, oxygen and silver expected for AgNPs/PG (Fig. 2C,E).

After that, the nano/microstructures AgNPs/PG was functionalized with magnetite nanoparticles (Fe_3_O_4_NPs) synthesized using coprecipitation method. SEM measurements and EDS analysis show that the functionalization with AgNPs and Fe_3_O_4_NPs of pollen grains was successful and don’t show changes on morphology of the pollen grain (Fig. 2B); also, the mapping show an homogenous distribution of the AgNPs and Fe_3_O_4_NPs under the template and he presence of the elements carbon, oxygen, iron and silver expected for hybrid nano/microstructures AgNPs-Fe_3_O_4_NPs/PG (Fig. 2D,F). The magnetite nanoparticles form agglomerates preferably in the valleys of the pollen microstructure (Fig. 1). The adhesion of the magnetic nanoparticles to AgNPs/PG composite can be tuned, through the combination of tailorable short-range interactions, an intermediate-range capillary force, and long-range magnetic attraction^10^.

**Figure 3:**
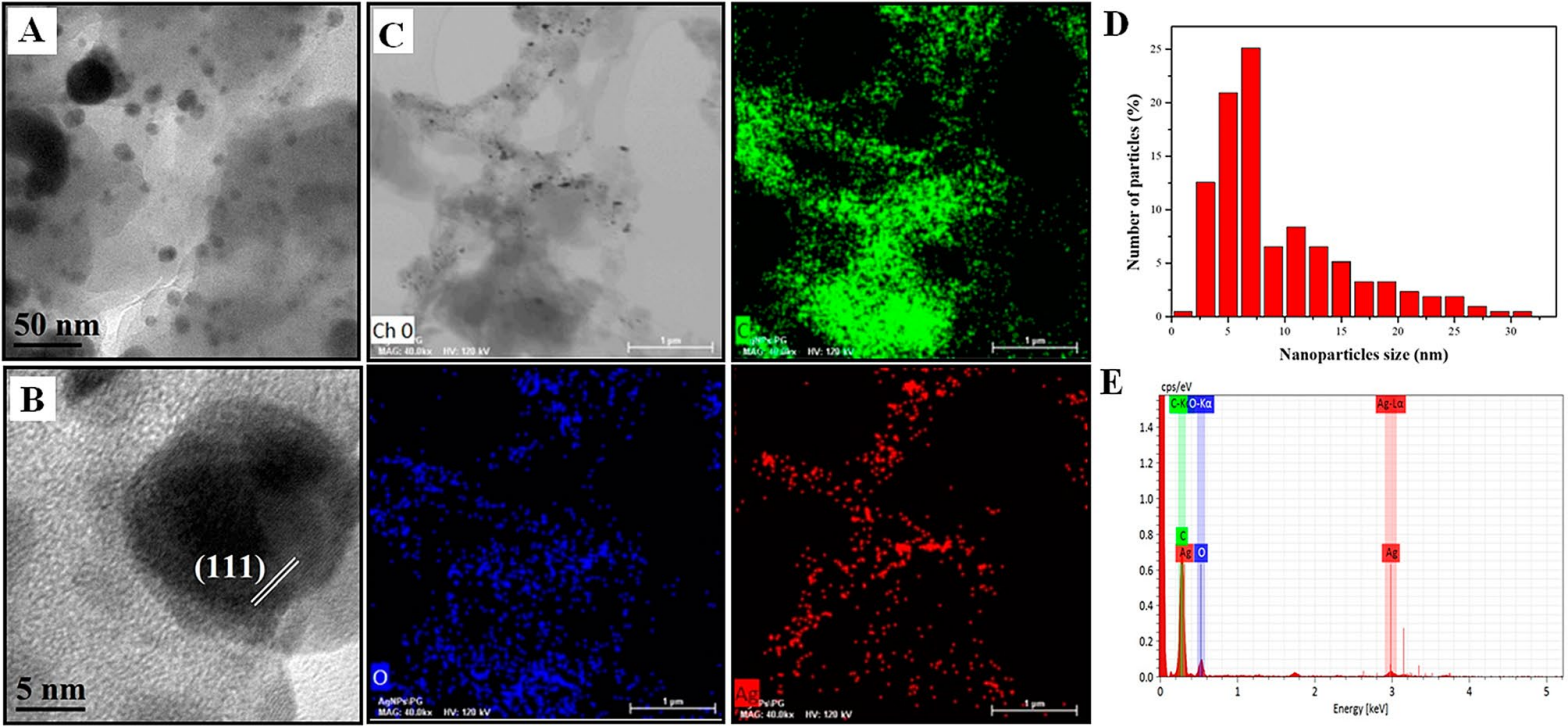
TEM Characterization of nano/microstructures AgNPs/PG. **(A)** TEM micrograph, **(B)** HRTEM micrograph and **(D)** histogram of size distribution of AgNPs; the Bragg reflections are indicated inside the figure corresponding to silver lattice spaces of (111). **(C)** Mapping of element distribution and **(E)** EDS analysis of nano/microstructures AgNPs/PG.

Representative biogenic AgNPs are shown in Fig. 3A with an average size of 9.37 ± 6.10 nm (Fig. 3D) and semispherical morphology. The lattice spacing in Fig. 3B was about 2.35 ± 0.02 Å between (111) planes consistent with FCC structure of silver. EDS mapping (Fig. 3C) and EDS spectrum (Fig. 3E) confirm the presence and homogenous distribution of AgNPs on the the exine of *Dimorphotheca ecklonis* pollen grains.

**Figure 4:**
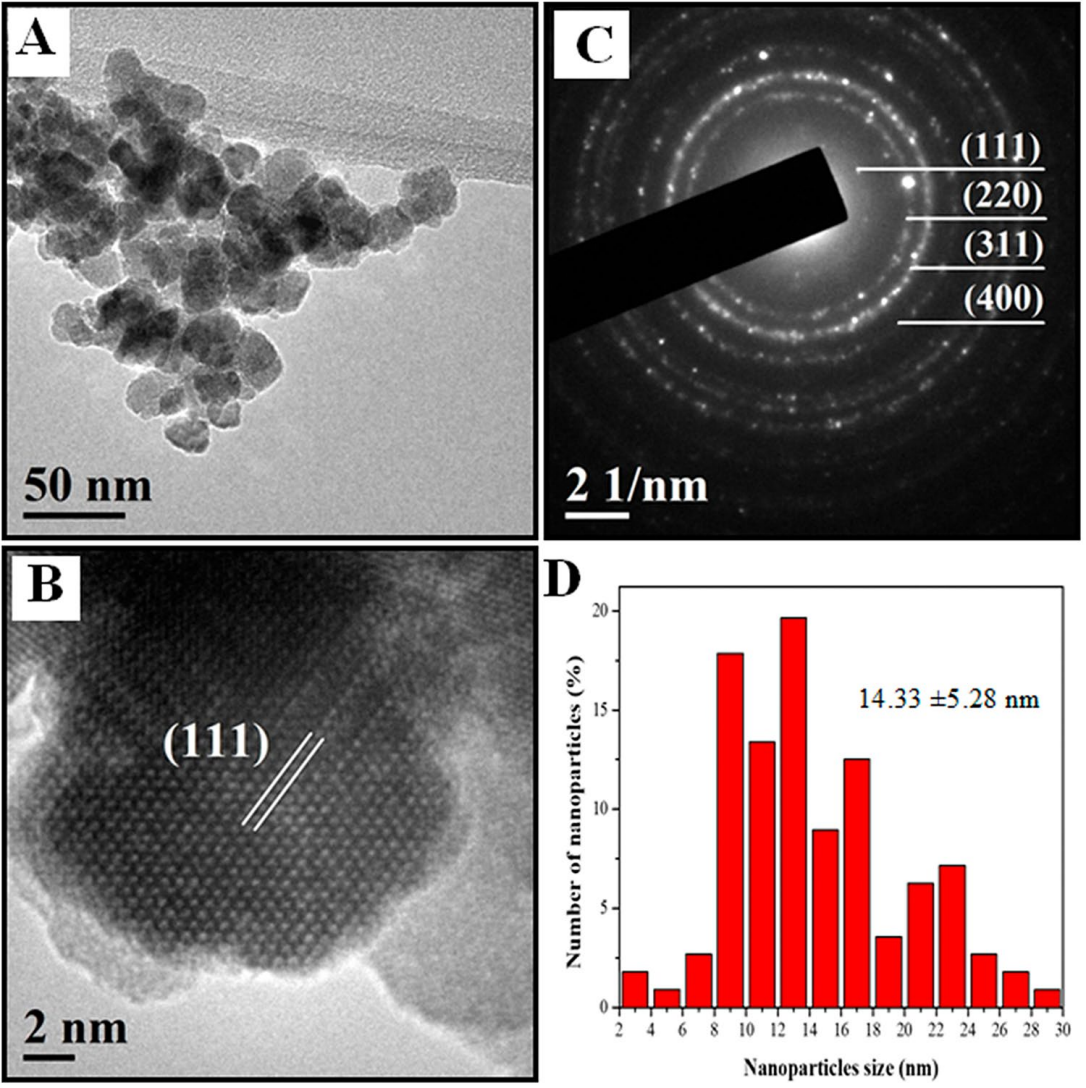
TEM Characterization of nano/microstructures Fe_3_O_4_ NPs. **(A)** TEM micrograph, **(B)** HRTEM micrograph, **(C)** SAED pattern, and **(D)** histogram of size distribution; planar distance and the bragg reflections are indicated inside each figure, respectively, corresponding to Fe_3_O_4_ lattice spaces of crystallography (111), (220), (311) and (422).

Fe_3_O_4_ nanoparticle synthesis was mediated by precipitation method, that’s produce spheroid nanoparticles with average size of 14.33 ± 5.28 nm, and range of most abundant between 12–14 nm (Fig. 4A,D); also, the average on planar distance 4.88 Å, 2.84 Å, 2.50 Å, and 1.59 Å for planar crystallography (111), (220), (311), (422) respectably (Fig. 4B,C); as were showed by TEM analysis.

**Figure 5:**
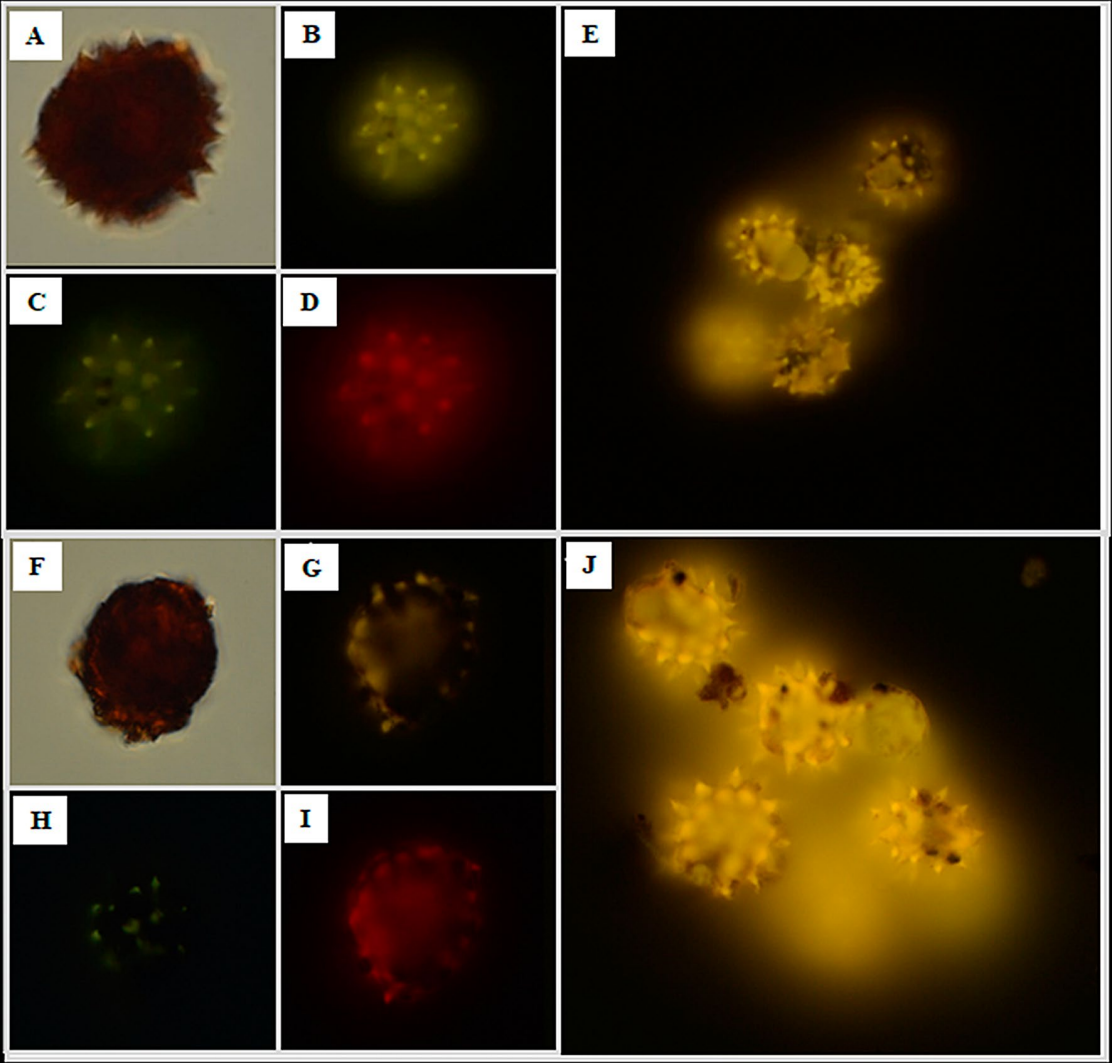
Fluorescence micrographs of hybrid nano/microstructures. **(A)** AgNPs/GP in the field clear, **(B)** yellow filter, **(C)** DAPI, **(D)** red, **(E)** cumulus yellow. **(F)** AgNPs-Fe_3_O_4_NPs/PG in the field clear, **(G)** yellow filter, **(H)** DAPI, **(I)** red, and **(J)** cumulus yellow.

On the other hand, the fluorescence property of the nano/microstructure were evaluating by confocal microscopy using a mercury lamp for fluorescence; showing the nano/microstructure AgNPs/PG and hybrid nano/microstructure AgNPs-Fe_3_O_4_NPs/PG considering fluorescence (Fig. 5). In general, AgNPs-Fe_3_O_4_NPs/PG (Fig. 5F–J) showed slower intensity of fluorescence that AgNPs/PG (Fig. 5A–E) in all acquisition channels; that suggest that this property is attributed to only to AgNPs deposited on the pollen grain and the Fe_3_O_4_NPs decrees the fluorescence blocking the AgNPs excitation.

**Figure 6:**
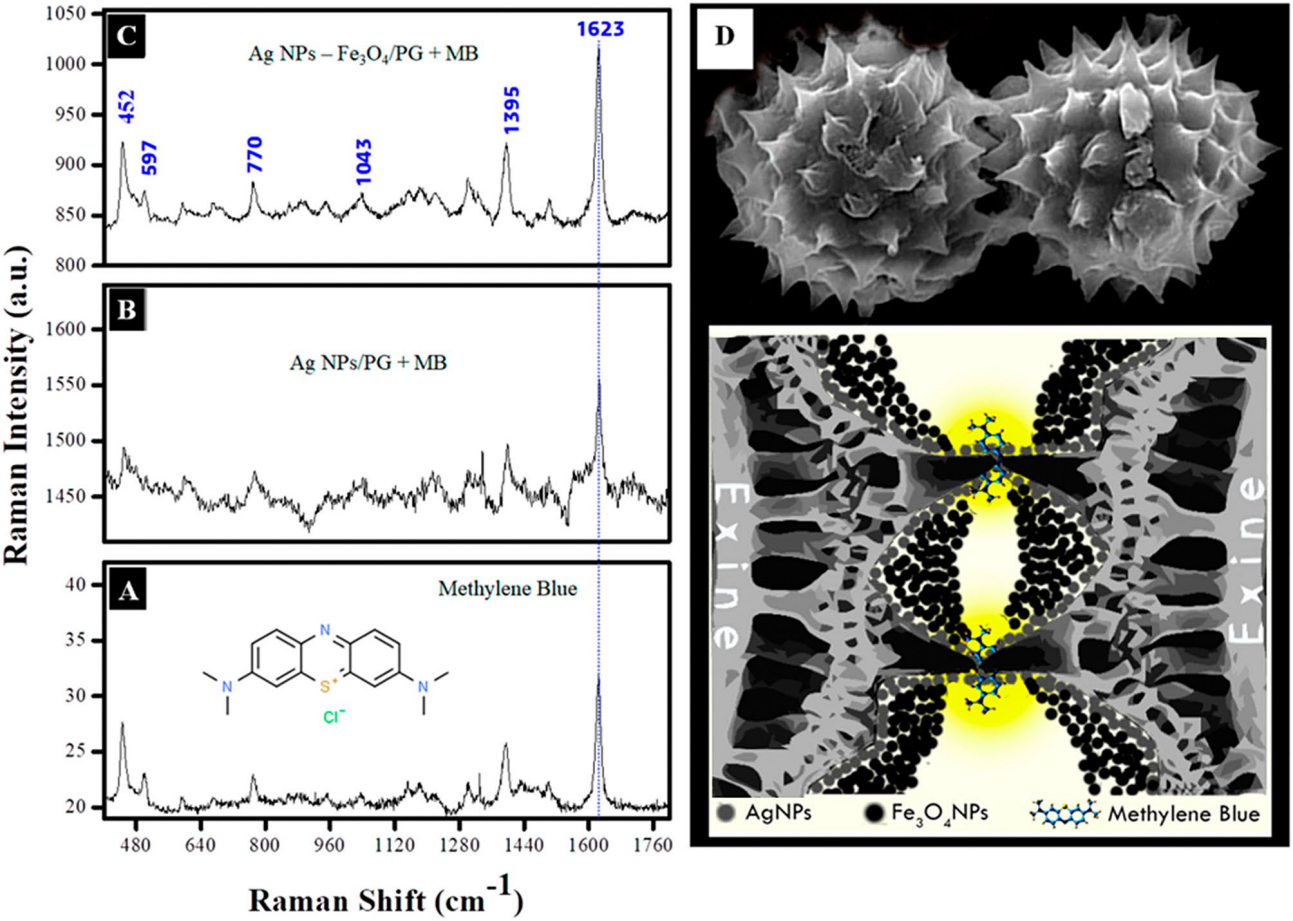
SERS Raman spectra of **(A)** methylene blue (MB), **(B)** MB + AgNPs/PG, and **(C)** MB + AgNPs-Fe_3_O_4_NPs/PG. **(D)** Schematic diagram of possible hot-spots generation in AgNPs-Fe_3_O_4_NPs/PG.

The hybrid nano/microstructures AgNPs/PG and AgNPs-Fe_3_O_4_NPs/PG were used as microscale SERS substrates, Raman spectra test was the evidence to enhance Raman signal of Methylene Blue, this was used as the probe molecule in this study since it has been well characterized in the literature. Considering the intensity peak of 1623.83 cm^−1^ as reference, we evaluate the ability of enhance Raman signal by the next equation:

$$EF=\frac{{I}_{NC}}{{I}_{MB}}=\frac{{I}_{pNC}-{I}_{blNC}}{{I}_{pMB}-{I}_{blMB}}$$

where EF is the enhancement factor, ${I}_{MB}$ is the intensity of the MB Raman spectra, ${I}_{NC}$ is the intensity of the MB Raman spectra using the hybrid nano/microstructure to evaluated as SERS surface, both obtained by ${I}_{p}$ (intensity of the peak) and ${I}_{bl}$(intensity of the base line). Considering this the EF for the nano/microstructures AgNPs/PG was 6.9596 and for hybrid nano/microstructures AgNPs-Fe_3_O_4_NPs/PG was 14.6969 (Fig. 6A–C).

The peak at 444 cm^-1^ and the peak at 499 cm^-1^ corresponds to skeletal deformation vibrations of C–N–C and C–S–C respectively^31,33^. 593 cm^-1^ peaks corresponding to C–N–C skeletal deformation^31,32^. C–H out-of-plane bending induce 670 cm^-1^ peaks^31,33^. 769 cm^-1^ peaks is generated by C–N–C and C–S–C skeletal deformation, also by N-CH_3_ stretching^34^. The presence of the 1040 cm^-1^ peak correlate to C–H in-plane. For the 1396 cm^-1^ peak, the C–H in plane ring deformation would induce its appearance^32^. On the other hand, 1500 cm^-1^ and 1623 cm^-1^ peaks are generated by C–C–C asymmetric skeletal deformation^35,36^ and C–C ring stretching^34,35^, respectively. An overview of the vibrational modes of MB corresponding to its Raman spectrum is shown in Table 1.

**Table 1:** Methylene blue (MB) vibration modes corresponding to its Raman spectrum, and spectra of SERS analysis using AgNPs/PG, and AgNPs-Fe_3_O_4_NPs/PG. Abbreviation: α, deformation of the ring in the plane; β, flexion in the plane; γ, flexion outside the plane; δ, skeletal deformation; and u, extension.

MB	MB + AgNPs/PG	MB + AgNPs-Fe_3_O_4_NPs/PG	Reference	Band Assignment
446	450	452	480^I^, 445^II^. 449^III^	δ(C–N–C), δ(C–N–C), δ(C–S–C)
502	–	508	497^II^, 502^III^	δ(C–N–C)
597	600	597	613^I^	δ(C–N–C)
670	664	670	677^II^, 688^III^	γ(C–H)
775	776	770	769^III^	δ(C–N–C), δ(C–S–C), ν(N-CH3)
882	–	895	895^VI^	
1032	1028	1043	1032^I^, 1036^II^, 1003^III^	β(C–H)
1395	1402	1395	1396^II^	α(C–H)
1501	1494	1507	1512^V^	δ _asym_ (C–C–C)
1623	1630	1623	1618^II^, 1617^IV^	ν(C–C) ring

I^31^, II^32^, III^33^, IV^34^, V^35^, VI^36^.

Comparing the SERS response of the nano/microstructures AgNPs/PG and the hybrid material AgNPs-Fe_3_O_4_NPs/PG; it is observed that an increase in the reference signal (1623.83 cm^-1^) and the MB Raman spectrum, it is more defined in the spectrum of the hybrid material. This phenomenon can be attributed to the distribution of nanoparticles on the pollen surface. In the AgNPs/PG material, the silver nanoparticles can be seen to be preferably at the tips of the pollen microstructure and to a lesser extent in the valleys; therefore, no hot-spots are generated that may favor the SERS response. In the hybrid material, the magnetite nanoparticles form agglomerates preferably in the valleys of the pollen microstructure, which causes the pollen grains to attract each other, favoring the approach of the pollen tips decorated with the silver nanoparticles, generating hot-spots, which are responsible for increasing the SERS signal (Fig. 6D).

## Conclusion

The pollen grains of *Dimorphotheca ecklonis* have been shown to be a good reducing and stabilizing agent in the synthesis of silver nanoparticles, as well as a biotemplete; the methodology that is used for these syntheses is adapted to the efficient synthesis and is more ecological that avoids the use of complex methodologies and the use of compounds and conditions of greater care, in contrast with that reported in the literature. In addition, the synthesis of magnetite nanoparticles by means of presetting method proved to be an efficient method to promote the synthesis, also the interaction between magnetite nanoparticles and hybrid nano/microstructures AgNPs/PG allows the functionalization with these nanoparticles and is dependent on the interaction time. Furthermore, SERS properties of nano/microstructures AgNPs-Fe_3_O_4_NPs based on *Dimorphotheca ecklonis* pollen grains were demonstrated by Raman analysis using methylene blue as a molecular signal, showing an enhancement factor of 14.6969; suggesting that in SERS not only the nature of the nanoparticles or the sizes and shape of the same, are important to improve the signal, but the disposition of the nanoparticles on the surface as well as the structure of the template can improve the SERS activity. Showing the ability to the methodology suggest here to produce a magnetic fluorescent hybrid nano/microstructure able to be a candidate as a pollen-microparticles sensor.

## References

[1] Makisima A (2004). Possibility of hybrid materials. Cer. Jap..

[2] Uemura, M. *Hybrid Composites* 1–9. (ed. Uemura, Hukunda, H.). (CMC Publishing Co., Tokyo, 2002)

[3] Nanko, M. Definitions and categories of hybrid materials. *Adv. In Tech. Mat. Mat. Proc. J.* **11**, 1–8 (2009)

[4] Wang Y, Liu Z, Han B, Huang Y, Yang G (2005). Carbon microspheres with supported silver nanoparticles prepared from pollen grains. Lang..

[5] McNally H, Pingle M, Lee S, Gou D, Bergstrom D, Bashir R (2003). Self-assembly of micro- and nano-scale particles using bio-inspired events. Appl. Surf. Sci..

[6] Goodwin W, Gomez I, Fang Y (2013). Conversion of pollen particles into three-dimensional ceramic replicas tailored for multimodal adhesion. Chem Mater..

[7] Scott R (1994). Pollen exine—The sporopollenin enigma and the physics of pattern. Soc. Exp. Biol. Semin. S..

[8] Blackmore S, Wortley A, Skvarla J, Rowley J (2007). Pollen wall development in flowering plants. New Phytol..

[9] Shen W, Zhang L, Du Y, Zhao B, Zhou X (2017). Synthesis, characterization, and properties of porous silver spheres using rape pollen as novel bio-templates. Mat. Lett..

[10] Johnstone L, Gomez I, Lin H, Fadiran O, Chen V, Meredith C, Perry J (2017). Adhesion enhancements and SERS activity of Ag and Ag@SiO2 nanoparticle decorated ragweed pollen micro-particle sensor. ACS Appl. Mater. Interfaces..

[11] Lin, H., Allen, M., Wu. J., deGlee, B., Shin, D., Cai, Y., Sandhage, K., Deheyn, D., Meredith, C. Dioenabled core/shell microparticles with tailored multimodal adhesion and optical reflectivity. *Chem. Mater*. **2015**. (2015)

[12] Gomez I, Goodwin W, Sbo D, Zhang Z, Sandhage K, Meredith J (2015). Three-dimensional magnetite replicas of pollen particles with tailorable and predictable multimodal adhesión. J. Mater. Chem. C..

[13] Laura M (2010). Metabolic response to cold and freezing of Osteospermum ecklonis overexpressing Osmyb4. Plant Phys. Biochem..

[14] Meier-Melikyan NR, Gabaraeva NI, Polevova SV (2003). Development of pollen grain walls and accumulation of sporopollenin. Russ. J. Plant Physiol..

[15] Kneipp K, Kneipp H, Itzkan I, Dasari RR, Feld MS (1999). Ultrasensitive chemical analysis by Raman spectroscopy. Chem. Rev..

[16] Kneipp, K., Wang, Y., Kneipp, H., Perelman, L. T., Itzkan, I., Dasari, R. R., Feld, M. S. Single molecule detection using surface-enhanced Raman scattering (SERS). *Phys. Rev. Lett.* **78**, 1667–1670 (1997)

[17] Wu D-Y, Li J-F, Ren B, Tian Z-Q (2008). Electrochemical surface-enhanced Raman spectroscopy of nanostructures. Chem. Soc. Rev..

[18] Qian X, Peng X-H, Ansari DO, Yin-Goen Q, Chen GZ, Shin DM, Yang L, Young AN, Wang MD, Nie S (2008). In vivo tumor targeting and spectroscopic detection with surface-enhanced Raman nanoparticle tags. Nat. Biotechnol..

[19] Qin L, Zou S, Xue C, Atkinson A, Schatz GC, Mirkin CA (2006). Designing, fabricating, and imaging Raman hot spots. Proc. Nat. Acad. Sci. U.S.A..

[20] Tripathi, K., Castro, M., Feller, J., Sonkar, S. Characterization of metal, semiconductor, and metal-semiconductor core–shell nanostructures. in *Micro and Nano Technologies, Metal Semiconductor Core-Shell Nanostructures for Energy and Environmental Applications*. 52–77 (2017).

[21] Chen W, Shi H, Wan F, Wang P, Gu Z, Li W, Ke LL, Huang Y (2017). Substrate influence on the polarization dependence of SERS in crossed metal nanowires. J. Mater. Chem. C..

[22] Lin W, Liao L, Chen Y (2011). Size dependence of nanoparticle-SERS enhancement from silver film over nanosphere (AgFON) substrate. Plasmonics.

[23] Kottmann J, Martin O, Smith D, Schultz S (2000). Spectral response of plasmon resonant nanoparticles with a non-regular shape. Opt Express..

[24] Noguez C (2007). Surface plasmons on metal nanoparticles: The influence of shape and physical environment. J. Phys. Chem. C..

[25] Blackmore S, Wortley A, Skvarla J, Gabarayeva N, Rowle J (2010). Developmental origins of structural diversity in pollen walls of compositae. Plant Syst. Evol..

[26] Iravani S (2011). Green synthesis of metal nanoparticles using plants. Green Chem..

[27] Verma SK, Jha E, Panda PK, Mishra A, Thirumurugan A, Das B, Parashar S, Suar M (2018). Rapid novel facile biosynthesized silver nanoparticles from bacterial release induce biogenicity and concentration dependent in vivo cytotoxicity with embryonic zebrafish–A mechanistic insight. Toxicol. Sci..

[28] Verma S.K., Jha E., Panda P.K., Thirumurugan A., Suar M. Biological effects of green-synthesized metal nanoparticles: A mechanistic view of antibacterial activity and cytotoxicity. in (eds Naushad, M., Rajendran, S., Gracia, F.) *Advanced Nanostructured Materials for Environmental Remediation. Environmental Chemistry for a Sustainable World* Vol. 25 (2019)

[29] Kanwal Z, Raza MA, Riaz S, Manzoor S, Tayyeb A, Sajid I, Naseem S (2019). Synthesis and characterization of silver nanoparticle-decorated cobalt nanocomposites (Co@AgNPs) and their density-dependent antibacterial activity. R. Soc. Open Sci..

[30] Patel, P., Kumari, P., Verma. S., Mallick, M. in *Cellular and Molecular Impact of Green Synthesized Silver Nanoparticles. Silver Nanoparticles-Health and Safety. IntechOpen*. 1–15 (2019)

[31] Ruan C, Eres G, Wang W, Zhang Z, Gu B (2007). Controlled fabrication of nanopillar arrays as active substrates for surface-enhanced Raman spectroscopy. Langmuir.

[32] Xiao GN, Man SQ (2007). Surface-enhanced Raman scattering of methylene blue adsorbed on cap-shaped silver nanoparticles. Chem. Phys. Lett..

[33] Zhong, L., Hu, Y., Xing, D. Adsorption orientation of methylene blue (MB+) on the silver colloids: SERS and DFT studies. in *CLEO/ Pacific Rim Conference* Vol. 978(2009)

[34] Virdee HR, Hester RE (1988). Surface-enhanced Raman spectroscopy of thinine-modified gold electrodes. Laser Chem..

[35] Xu, W., Aydin, M., Zakia, S., Atkins, D.L. Aggregation of thionine within AIMCM-48. *J. Phys. Chem. B.* **108**, 5588–5593 (2004)

[36] Quester K, Avalos-Borja M, Vilchis-Nestor AR, Camacho-Lopez MC, Castro-Longoria E (2013). SERS Proprieties of different sized and shaped gold nanoparticles biosynthesized under different environmental conditions by Neurospora crassa extract. PLoS ONE.

